# RES5/464: OsTd-Online - A System for Web-Based Standardised and Organ Specific Tumor Documentation

**DOI:** 10.2196/jmir.1.suppl1.e80

**Published:** 1999-09-19

**Authors:** A Wolff, P Knaup, J Finnern, P Hermanek, G Wagner, KH Ellsässer, R Haux

**Affiliations:** ^1^University of Heidelberg, HeidelbergGermany

**Keywords:** Tumor Documentation, Standardised Documentation, Documentation Systems, Clinical Trials, Guidelines

## Abstract

**Introduction:**

The multitude of different tumor diseases, each of them having its own special and extensive procedures, requires documentation guidelines for clinical oncologists. One of these is the organ specific tumor documentation from G. Wagner and P. Hermanek (1995) which at present is updated and extended. The first edition embraces 36 chapters for the most common tumor diseases and two chapters for lung respectively liver metastasises. Each chapter consists of a list of questions about pre-therapeutical data, data concerning therapy and data concerning pathology. Additionally, there are special coding instructions for questions which need further explanation. Because of the advantages of having data not only written down on sheets of paper but stored in a database the new edition of the organ specific tumor documentation will only be delivered as electronic version. Therefore, a web-based documentation system called OsTd-Online is developed by the University of Heidelberg (Department of Medical Informatics) in cooperation with the German Cancer Society. OsTd-Online will cover all 43 chapters of the second edition and offer standardised case report forms for cancer patients in dependency of the kind of tumor.

**Methods:**

Each chapter contains several case report forms built in HTML 4.0. Via JDBC-Connection the data could be inserted into an Access-Database. Documenting is supported by hyperlinks providing context-sensitive help (e. g. references to coding instructions or bibliography).Cascading Style Sheets (CSS1) are used to facilitate uniform design. CSS is also utilized in combination with JavaScript 1.2 procedures to show respectively hide items that depend on the value of another item (e. g. the item 'surgeon' should only be displayed if the value of the item 'operation' is set on 'yes'). Moreover plausibility checks and special printing functions are implemented by JavaScript.

**Results:**

The project is still under development. However, in September 1999 a prototype with full functionality will be finished including chapters for documenting Radiotherapy, Chemotherapy and Progress of a patient as well as four chapters about organ specific tumor diseases. At the beginning of next year a CD-ROM with the first reduced version of OsTd-Online including about 15 organ specific chapters will be published.

**Discussion:**

A standardised tumor documentation is a prerequisite for quality assurance and statistical research especially in multi-center clinical trials. By making OsTd-Online easy accessible for every interested physician we hope to motivate more physicians to document more carefully. Besides this OsTd-Online should improve the quality of documentation by automatic verification to increase correctness, completeness and consistency of documented data.

